# Supplementary material for: What supports and constrains the implementation of multifactorial falls risk assessment and tailored multifactorial falls prevention interventions in acute hospitals? Protocol for a realist review
Source: BMJ Open. 2021 Sep 2;11(9):e049765. doi: 10.1136/bmjopen-2021-049765 (PMC8413962; doi:10.1136/bmjopen-2021-049765)
Supplement: Supplementary data [file bmjopen-2021-049765supp002.pdf]

What supports and constrains the implementation of multifactorial falls risk assessment and tailored multifactorial falls prevention interventions in acute hospitals? Protocol for a realist review (Randell et al.)

Additional file 2: Practitioner Theories Search Strategy Example

Ovid MEDLINE(R) and Epub Ahead of Print, In-Process & Other Non-Indexed Citations and Daily <1946 to July 22, 2020>

1 Accidental Falls/ or exp Hip Fractures/pc (25502)
2 (fall or falls or faller*).tw,kw. (147405)
3 or/1-2 [falls] (155967)
4 Risk Assessment/ (265290)
5 risk assess*.tw,kw. (69279)
6 (fall* adj3 (assess* or screen* or prevent* or predict*)).tw,kw. (10727)
7 exp Accident Prevention/ (86820)
8 or/4-7 [assessment or prevention] (391878)
9 nursing time*.jn. (39139)
10 3 and 8 and 9 (26)
11 nursing standard.jn. (35276)
12 3 and 8 and 11 (33)
13 health service* journal.jn. (10933)
14 3 and 8 and 13 (3)
15 10 or 12 or 14 (62)
