# Supplementary material for: What supports and constrains the implementation of multifactorial falls risk assessment and tailored multifactorial falls prevention interventions in acute hospitals? Protocol for a realist review
Source: BMJ Open. 2021 Sep 2;11(9):e049765. doi: 10.1136/bmjopen-2021-049765 (PMC8413962; doi:10.1136/bmjopen-2021-049765)
Supplement: Supplementary data [file bmjopen-2021-049765supp003.pdf]

What supports and constrains the implementation of multifactorial falls risk assessment and tailored multifactorial falls prevention interventions in acute hospitals? Protocol for a realist review (Randell et al.)

Additional file 3: Academic Theories Search Strategy Example

Ovid MEDLINE(R) and Epub Ahead of Print, In-Process & Other Non-Indexed Citations and Daily <1946 to July 21, 2020>

1 Accidental Falls/ or exp Hip Fractures/pc (25500)
2 (fall or falls or faller*).tw,kw. (147448)
3 or/1-2 [falls] (156010)
4 Risk Assessment/ (265251)
5 risk assess*.tw,kw. (69315)
6 (fall* adj3 (assess* or screen* or prevent* or predict*)).tw,kw. (10733)
7 exp Accident Prevention/ (86806)
8 or/4-7 [assessment or prevention] (391875)
9 Hospitalization/ (107412)
10 Subacute Care/ (1049)
11 Hospital Units/ (10146)
12 exp Hospitals/ (274581)
13 Rehabilitation Centers/ (8183)
14 Inpatients/ (21949)
15 ((acute or sub-acute or subacute) adj3 (care or ward?)).tw,kw. (31737)
16 ((rehabilitation or geriatric) adj (ward? or unit? or department?)).tw,kw. (6423)
17 inpatient?.tw,kw. (107879)
18 hospital*.tw,kw. (1294309)
19 or/9-18 [hospital] (1485504)
20 3 and 8 and 19 [Fall assmt & prevention in hospitals] (3313)
21 meta-analysis/ or "systematic review"/ (194072)
22 (Literature review* or (systematic adj2 review*) or (narrative adj2 review*) or (critical adj2 review*) or scoping review* or synthesis or meta-analys* or "meta analysis" or (realist adj2 review*)).ti. (551543)
23 ("Search filter*" or "search strateg*" or "literature search*").ab. (66610)
24 or/21-23 [Systematic reviews] (635237)
25 20 and 24 (139)
